# A rare case of absolute thrombocytopaenia in a COVID-19 patient: Case report

**DOI:** 10.1016/j.amsu.2021.103097

**Published:** 2021-11-22

**Authors:** Ameen M. Mohammad, Azri S.H. Sgery, Nawfal R. Hussein

**Affiliations:** a Department of Medicine, College of Medicine, University of Duhok, Kurdistan Region, Iraq; b Medical Student at College of Medicine, University of Duhok, Kurdistan Region, Iraq; c Department of Biomedical Science, College of Medicine, University of Zakho, Kurdistan Region, Iraq

**Keywords:** COVID-19, SARS-CoV-2, Thrombocytopaenia, Immune thrombocytopaenia, Immune thrombocytopaenic purpura (ITP), Drug-induced immune thrombocytopaenia (DITP)

## Abstract

**Introduction:**

Thrombocytopaenia, one of the most common haematological disorders worldwide, is characterised by platelet counts <150,000/mm^3^. Patients with coronavirus disease (COVID-19) were found to commonly exhibit haematological abnormalities, often with mild forms of thrombocytopaenia. Absolute thrombocytopaenia tends to be rare among these patients and is believed to be secondary to immune-induced thrombocytopaenia.

**Case presentation:**

A 53-y-old man presented with fever and generalised body ache that persisted for a few days. His polymerase chain reaction test was positive for COVID-19, for which he was treated with acetaminophen, levofloxacin, and favipiravir. On the third day of treatment, he noticed bruising and bleeding, mainly in the oral cavity, with clot formation. A complete blood picture (CBP) revealed severe thrombocytopaenia with an almost-zero count. Prednisone 1 mg/kg/d and frequent doses of intravenous platelet transfusion were administered as rescue therapy to prevent fatal bleeding. The patient was able to recover.

**Clinical discussion:**

Immune thrombocytopaenia should be considered in patients presenting with bleeding tendencies after severe acute respiratory syndrome coronavirus 2 infection. Serial CBP is recommended for vulnerable patients, especially during the second and third weeks of hospitalisation, for the early detection and prevention of life-threatening COVID-19 complications.

**Conclusions:**

Absolute thrombocytopaenia is a rare condition. Such a condition should be considered in patients presenting with bleeding tendencies with severe Covid-19 infection. With early diagnosis and appropriate treatment, patients’ lives can be saved.

## Highlights

• Absolute thrombocytopaenia is a rare condition.
• Such a condition should be considered in patients presenting with bleeding tendencies with severe Covid-19 infection.
• Prednisone 1 mg/kg/d and intravenous platelet transfusion should be considered as rescue therapy to prevent fatal bleeding.

## 1 Introduction

Thrombocytopaenia, one of the most common haematological disorders, is characterized by a platelet count <150,000/mm^3^ with a broad variation of clinical manifestations, ranging from asymptomatic to life-threatening bleeding. Patients with thrombocytopaenia usually seek medical help when they suffer from spontaneous bruising, purpura, or other symptoms, which usually occur when the platelet count reaches <30,000/mm^3^ [1].

The novel severe acute respiratory syndrome coronavirus 2 (SARS-CoV-2) virus, which is responsible for coronavirus disease (COVID-19), is a serious public health concern worldwide with variable clinical manifestations [[2], [3], [4], [5]]. In the Kurdistan region of Iraq, we experienced three waves with devastating impacts on our health system [6,7]. With the emerging data, the virus was found to be involved in other systems, such as the vascular, gastrointestinal, and haematological systems [8].

COVID-19 patients commonly exhibit haematological abnormalities, often with mild forms of thrombocytopaenia and other haematological findings. The incidence of thrombocytopaenia in COVID-19 patients tends to be variable, with mild cases commonly observed in severe COVID-19 cases; however, absolute thrombocytopaenia with a count of almost zero tends to be very rare [9,10].

Increasingly, cases of immune-induced thrombocytopaenia have been reported in patients following their SARS-CoV-2 infection and are described as secondary immune thrombocytopenic purpura (ITP), or immune thrombocytopaenia in the absence of purpura, triggered by SARS-CoV-2 viral infection with the viral induction of autoimmunity, explained in terms of molecular mimicry, cryptic antigen expression, or epitope spreading. Hence, immune thrombocytopaenia has been reported as an important SARS-C0V-2 complication [11]. In this report, we present a case of severe thrombocytopaenia following SARS-CoV-2 infection.

## 2 Patient information

A 53-y-old male patient with no previous significant comorbidities presented to the hospital with a history of fever and generalised body ache that persisted for a few days. The polymerase chain reaction (PCR) test was positive for COVID-19, for which the patient started to receive treatment. On the third day of treatment, the patient noticed bruising and bleeding, mainly in the oral cavity, with the formation of blood clots inside the mouth. Laboratory investigation and complete blood picture (CBP) was performed, which revealed severe thrombocytopaenia with a count of almost zero. The patient was then treated for thrombocytopaenia to prevent fatal bleeding. Our case has been reported in line with THE SCARE 2020 criteria [12]. The clinical examination and all procedures were performed and supervised by two professors of internal medicine. The patient was fit and not receiving any regular medications. The family history was negative in respect to the disorder of the patient. The psychological history was negative. Written informed consent was obtained from the patient for publication of this case report and accompanying images. A copy of the written consent is available for review by the Editor-in-Chief of this journal on request.

### 2.1 Laboratory findings

On September 26, 2020, the patient tested positive for SARS-CoV-2 via real-time PCR (RT-PCR) using nasopharyngeal and oropharyngeal swabs. His CBP results indicated severe thrombocytopaenia with a platelet count of 2/mm^3^, or almost zero, 2 d after testing positive. Thrombocytopaenia persisted for another 4 d. On October 3, 2020, there was a dramatic increase in the platelet count, reaching 28,000/mm^3^, and by October 12th, it reached 89,000/mm^3^.

The absolute neutrophil count in the first laboratory findings was normal. A significant rise was noticed on the fifth day after the COVID-19 test and continued until October 12, 2020. Lymphocytic counts tended to fluctuate between normal, low, and high. On the other hand, the white blood cell count was high on the fifth day post-COVID-19 test and remained the same. Haemoglobin levels tended to be in the lower normal range.

On the fourth day post-COVID-19 test, the patient's laboratory findings showed normal D-dimer (330) partial thromboplastin time (29 s), international normalised ratio (1.0), S. creatinine (1.1), and alanine aminotransferase levels (43). While lactate dehydrogenase (618), S. ferritin (1722), and c-reactive protein (29.6) levels were high. The Coombs test was negative, and he had an (A-negative) blood type (see Table 1, Table 2).

**Table 1:** Laboratory findings of the patient after testing positive for SARS-CoV-2.

	Platelets (*1000/mm^3^)	WBC (*1000/mm^3^)	Hemoglobin (g/dl)	Lymphocyte (%) & Absolute count (per/UI)	Neutrophil (%) & Absolute count (per/UI)
28th/9/2020	2*	6.3	13.8	(26%) 1.64	(70%) 4.41
29th/9/2020	2*	4.9	13.5	(12%) 0.59*	(88%) 4.31
1st/10/2020	5*	12.5**	11.8	(7%) 875	(88%) 11000**
	3*	11.5**	12.3	(10%) 1.15	(88%)10.12**
3rd/10/2020	28	20**	13	(13%) 2600	(81%) 16200**
6th/10/2020	26	26.9**	13.2	(17%) 4573**	(81%) 21789**
12th/10/2020	89	14.7**	13.4	(12%) 1764	(87%) 12789**
14th/5/2021	188	12.4*	13.3	(25%) 3.1	(69%) 8.56**

Abbreviations: SARS-CoV-2, severe acute respiratory syndrome coronavirus 2; WBC, white blood cells.

**Table 2:** Laboratory findings on September 30, 2020.

D-Dimer	Blood Group	PTT	INR	S. Creatinine	ALT	LDH	S. Ferritin	Coombs Test	CRP
330	A -ve	29 sec.	1.0	1.1	43	618*	1722*	Negative	29.6*

Abbreviations: PTT, partial thromboplastin time; INR, international normalised ratio; ALT, alanine aminotransferase; LDH, lactate dehydrogenase; CRP, c-reactive protein.

### 2.2 Therapeutic intervention

The patient received treatment for COVID-19 after confirmation. This consisted of acetaminophen, levofloxacin tablets, and favipiravir. After the diagnosis of thrombocytopaenia, he was placed on a course of prednisone 1 mg/kg/day (70 mg) and frequent doses of intravenous platelet transfusion as rescue therapy to prevent fatal bleeding.

### 2.3 Follow-up and outcomes

After several days of severe thrombocytopaenia and a platelet count <50r/mm^3^, the patient showed a good response to the treatment, with a gradual rise in the platelet count. On the 16th day after testing positive, his platelet count reached 89,000/mm^3^, which was considered moderate thrombocytopaenia. The patient completely recovered from the bleeding tendency and manifestations. With an outpatient follow-up for 1 month, the patient was asymptomatic when he returned and CBP was completely normal.

## 3 Discussion

Despite the strict measures taken to prevent infection in our region, the number of COVID-19 cases has increased sharply, with a concurrent increase in severity and case-fatality rate [[13], [14], [15], [16]]. In this report, we present a case of COVID-19 with severe thrombocytopaenia and presentations of a history of fever and generalised body ache for a few days, followed by features of bleeding tendency during the course of COVID-19 treatment. SARS-CoV-2 has the potential to present with a spectrum of variable presentations and can involve one or more systems of the body. Due to the variability in the presentation, cases of thrombocytopaenia, despite being common, especially in severe cases of COVID-19, can pass unnoticed until it reaches severe forms and ends in patients developing complications, such as intracranial bleeding, gastrointestinal bleeding, and haemoptysis [10,11].

The three proposed mechanisms for COVID-19-associated thrombocytopaenia include a decrease in platelet production either directly due to bone marrow invasion and cytokine storm or indirectly due to lung injury, a decrease in circulating platelets due to increased platelet consumption, such as disseminated intravascular coagulopathy (DIC), and an increase in platelet destruction due to autoantibodies [9,17].

Thrombocytopaenia has an important prognostic value as lower platelet counts are associated with an increased risk of in-hospital mortality among COVID-19 patients; the lower the platelet count, the higher the risk. Clinical improvement was seen with the improvement of thrombocytopaenia in COVID-19 patients ( [10,18]. Thrombocytopaenia is usually multifactorial with a complex pathogenesis; however, in the current case with a platelet count of almost zero, immune-induced thrombocytopaenia is far more likely than other aetiologies [19].

In the current case of COVID-19, severe thrombocytopaenia was observed for several days, followed by a gradual increase in the platelet count after management with steroids and platelet transfusion. In cases of thrombocytopaenia with normal D-dimer levels, prothrombin time, and activated partial thromboplastin time, and without any features of haemolysis, immune thrombocytopaenia becomes likely with SARS-CoV-2-triggered auto-antibodies against platelets as the underlying mechanism [20].

In this case, the severe degree of thrombocytopaenia, which was possibly associated with a sudden drop >50% over 24–48 h, further supports the diagnosis of immune thrombocytopaenia [17]. Additionally, immune thrombocytopaenia has previously been described following several viral infections, including hepatitis B/C viruses, cytomegalovirus, varicella zoster virus, and, recently, COVID-19 [17,20].

Although the onset of immune thrombocytopaenia secondary to SARS-CoV-2 was commonly seen in the second and third week after COVID-19, the onset of this case within the first week could be attributed to the failure of the patient to recognise the onset of COVID-19 symptoms [17].

Drug-induced immune thrombocytopaenia due to levofloxacin could be regarded as a possible differential diagnosis because it presents with severe thrombocytopaenia [21]. However, we found no cases to support favipiravir-induced thrombocytopaenia in clinical practice. Additionally, with a normal D-dimer level, DIC was unlikely. Glucocorticoids and/or immunoglobulins are regarded as first-line treatment for severe secondary thrombocytopaenia, as they interfere with platelet destruction, while second-line treatments include thrombopoietin receptor agonists, rituximab, and splenectomy [20].

Recent studies have reported a significant correlation between COVID-19 and blood parameters, including platelets, and the severity of thrombocytopaenia seemed to be associated with the severity of the disease; patients with severe COVID-19 had a lower platelet count, according to a meta-analysis [8,10,19,22]. Variability in the presentation of COVID-19 leads to the under-diagnosis of thrombocytopaenia and ends in patients developing complications [11]; hence, a systemic approach will be necessary to identify thrombocytopaenia in patients with COVID-19 and to exclude other causes.

With recent reports showing cases of COVID-19-associated thrombocytopaenia [20], we recommend the following: any patient diagnosed with SARS-CoV-2 infection, especially moderate to severe cases, requires a serial laboratory check-up for platelet counts as thrombocytopaenia could be established prior to admission or during hospitalisation [11]. Conversely, patients presenting with the sole manifestation of thrombocytopaenia should be screened for SARS-CoV-2 infection.

## 4 Conclusion

In the current pandemic, thrombocytopaenia should be considered in patients presenting with bleeding tendencies after SARS-CoV-2 infection. SARS-CoV-2-triggered autoantibodies against platelets could be regarded as a possible mechanism behind very severe thrombocytopaenia in our patient. The treatment applied for thrombocytopaenia in the index case is principally the same as that for non-COVID-induced immune thrombocytopaenia. Our case above highlights the wide spectrum of clinical presentations of Covid-19 infection. We emphasize the importance of early recognition as the early diagnosis of such a condition is crucial for timely therapeutic intervention, improved survival, and reduced morbidity.

## Ethical approval

This study and the consent of the case report was approved by the Ethics Committee in The College of Medicine, University of Zakho, Kurdistan Region of Iraq.

## Sources of funding

None.

## Authors contribution

AMM and NRH diagnosed, managed and followed up the case. ASS collected the data and followed up the patient. AMM and NRH write the draft and all authors approved the final version.

## Trial registry number

None.

## Consent

Written informed consent was obtained from the patient for publication of this case report and accompanying images. A copy of the written consent is available for review by the Editor-in-Chief of this journal on request.

## Guarantor

Ameen Mosa Mohammad, Department of Medicine, College of Medicine, University of Duhok, Kurdistan region, Iraq. Email: doctoramb@yahoo.com.

## Provenance and peer review

Not commissioned, externally peer-reviewed.

## Declaration of competing interest

None.

## Abbreviations

COVID-19: coronavirus disease
SARS-CoV-2: severe acute respiratory syndrome coronavirus 2
WBC: white blood cells
PTT: partial thromboplastin time
INR: international normalised ratio
ALT: alanine aminotransferase
LDH: lactate dehydrogenase
CRP: c-reactive protein
DIC: disseminated intravascular coagulopathy
